# Facilitating evidence uptake: development and user testing of a systematic review summary format to inform public health decision-making in German-speaking countries

**DOI:** 10.1186/s12961-018-0307-z

**Published:** 2018-07-09

**Authors:** Laura K. Busert, Margot Mütsch, Christina Kien, Aline Flatz, Ursula Griebler, Manfred Wildner, Jan M. Stratil, Eva A. Rehfuess, on behalf of Cochrane Public Health Europe

**Affiliations:** 1 Institute for Medical Information Processing, Biometry and Epidemiology, Pettenkofer School of Public Health, Ludwig-Maximilians-Universität München, Marchioninistr. 15, 81377 Munich, Germany; 2 Epidemiology, Biostatistics and Prevention Institute, University of Zurich, Hirschengraben 84, 8001 Zurich, Switzerland; 3 Department for Evidence-based Medicine and Clinical Epidemiology, Danube University Krems, Dr.-Karl-Dorrek-Straße 30, 3500 Krems, Austria; 4 Cochrane Switzerland, Institute of Social and Preventive Medicine (IUMSP), Lausanne University Hospital, Biopôle 2, Route de la Corniche 10, 1010 Lausanne, Switzerland

**Keywords:** Systematic review, public health, decision-making, knowledge dissemination, knowledge translation, evidence synthesis, evidence-based public health, GRADE

## Abstract

**Background:**

Systematic reviews are an important source of evidence for public health decision-making, but length and technical jargon tend to hinder their use. In non-English speaking countries, inaccessibility of information in the native language often represents an additional barrier. In line with our vision to strengthen evidence-based public health in the German-speaking world, we developed a German language summary format for systematic reviews of public health interventions and undertook user-testing with public health decision-makers in Germany, Austria and Switzerland.

**Methods:**

We used several guiding principles and core elements identified from the literature to produce a prototype summary format and applied it to a Cochrane review on the impacts of changing portion and package sizes on selection and consumption of food, alcohol and tobacco. Following a pre-test in each of the three countries, we carried out 18 user tests with public health decision-makers in Germany, Austria and Switzerland using the ‘think-aloud’ method. We analysed participants’ comments according to the facets credibility, usability, understandability, usefulness, desirability, findability, identification and accessibility. We also identified elements that hindered the facile and satisfying use of the summary format, and revised it based on participants’ feedback.

**Results:**

The summary format was well-received; participants particularly appreciated receiving information in their own language. They generally found the summary format useful and a credible source of information, but also signalled several barriers to a positive user experience such as an information-dense structure and difficulties with understanding statistical terms. Many of the identified challenges were addressed through modifications of the summary format, in particular by allowing for flexible length, placing more emphasis on key messages and relevance for public health practice, expanding the interpretation aid for statistical findings, providing a glossary of technical terms, and only including graphical GRADE ratings. Some barriers to uptake, notably the participants’ wish for actionable recommendations and contextual information, could not be addressed.

**Conclusions:**

Participants welcomed the initiative, but user tests also revealed their problems with understanding and interpreting the findings summarised in our prototype format. The revised summary format will be used to communicate the results of Cochrane reviews of public health interventions.

**Electronic supplementary material:**

The online version of this article (10.1186/s12961-018-0307-z) contains supplementary material, which is available to authorized users.

## Background

Systematic reviews that identify, appraise and synthesise all available research findings in relation to a specific research question constitute an important source of evidence for public health decision-making. Cochrane has become the world’s largest producer of systematic reviews of health interventions and, within this network, Cochrane Public Health is specifically concerned with the effects of population-level health interventions that address the structural and social determinants of health. Cochrane Public Health Europe (CPHE) is the European satellite of Cochrane Public Health [1, 2]. In line with the recently launched Cochrane Collaboration’s Knowledge Translation Strategy [3], one of CPHE’s aims is to support the dissemination of evidence from Cochrane reviews in the European region and to facilitate an increased and more rapid uptake of findings in public health policy and practice.

Several studies have examined factors that influence the use of evidence in policy-making [4–9]. The literature tends to distinguish between ‘push’ activities undertaken by research organisations to disseminate research evidence, ‘pull’ activities undertaken by decision-makers to access and use research evidence, and ‘exchange’ activities to build and maintain relationships between researchers and decision-makers [10–13]. Among these activities, decision-makers usually prioritise formal or informal exchange efforts to support evidence-informed decision-making [11]. Furthermore, decision-makers benefit from having highlighted information that is relevant for them and their specific decision-making context (e.g. contextual factors that affect local applicability) and from having systematic reviews presented in a way that allows for rapid scanning for relevance and a graded entry (e.g. key messages followed by a short summary) [14, 15]. Factors that inhibit the use of systematic reviews are the length of articles, use of jargon and exclusive publishing for a scholarly audience in academic journals [14, 15]. A systematic review of the effectiveness of systematic review summaries in increasing policy-makers’ use of such evidence concluded that summaries are somewhat easier to understand than full reviews [16].

In non-English speaking countries, the language of the full systematic reviews often constitutes an additional barrier to accessing evidence, which Cochrane attempts to overcome by providing translations of abstracts and Plain Language Summaries of Cochrane reviews in multiple languages. With ‘Cochrane Kompakt’ [17], Cochrane groups in the three German-speaking countries undertake and promote the translation of selected up-to-date Plain Language Summaries into German. We are not aware of any other concerted effort to promote the uptake of evidence from systematic reviews in the German language.

In line with our vision to strengthen evidence-based public health in the German-speaking countries and elsewhere and in working towards an increased and more rapid uptake of findings in public health policy and practice, CPHE member institutions joined forces to develop a German language summary format for systematic reviews of public health interventions. Our objectives were to develop a summary format suitable for public health decision-makers in Germany, Austria and Switzerland, and to undertake formal user-testing. While our initial focus was on Cochrane Public Health reviews, ultimately, the summary format should be applicable to Cochrane as well as non-Cochrane reviews of public health interventions.

## Methods

**Fig. 1:**
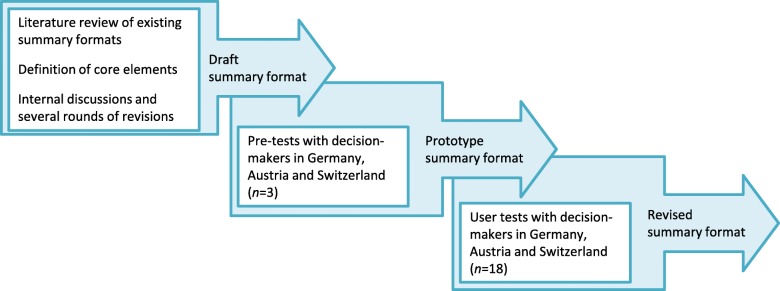
Process for the development and testing of the summary format

Ethical approval was granted by the Ethics Committee of the Ludwig-Maximilians-Universität München (Munich, Germany), and responsibility waived by the Ethics Committee of the University for Continuing Education (Krems, Austria) and the Ethics Committee Zurich (Zurich, Switzerland). The protocol for this study is available from the corresponding author upon request. Figure 1 outlines the process for developing and testing the summary format.

### Development of the prototype summary format

To inform the development process, we searched the literature for existing tools for summarising the findings of systematic reviews in the health field. An inventory of the tools we identified and their characteristics is provided in Additional file 1: Table S1; we did not find a tool specifically developed for public health interventions. Within the research team we decided on guiding principles and core elements to be included in the summary format (Fig. 1).

#### Overall structure

The overall structure of the summary format was guided by the so-called ‘1–3–25’ strategy developed and used by the Canadian Health Services Research Foundation [18]. This strategy proposes a one-page outline of the key messages, a three-page executive summary and a 25-page complete research report. For our purposes, we set out to implement the ‘1–3’ part and to produce a summary format of four pages. The full systematic review represents the ‘25’ part, even though full systematic reviews, especially Cochrane reviews, are often significantly longer.

#### Elements specific to systematic reviews

Given its routine use in Cochrane reviews, we decided to include the Summary of Findings (SoF) table to present the main results. This table was critically informed by the Grading of Recommendations Assessment, Development and Evaluation (GRADE) approach to assess the certainty of evidence [19]. In addition to the GRADE rating (i.e. high, moderate, low or very low certainty of evidence), the SoF table also provides information concerning the magnitude of effect of the interventions examined and the available data on all important outcomes for a given comparison. In those instances, where the SoF table was not considered the best way to present findings or where information provided in this manner was considered insufficient, text was also used.

#### Public health-relevant aspects

Public health interventions are often highly context dependent [20] and public health decision-makers are thus concerned with factors that may influence the local applicability of findings from systematic reviews. Relevant factors include the specific context in which individual studies were conducted (e.g. setting, country), feasibility of the intervention, acceptability, equity considerations, differential effects in subgroups, risks associated with the intervention, resource use (costs, intervention providers) and value for money [14, 21–23]. One section of the summary format provides space for these aspects to be addressed.

Based on these guiding principles and core elements, we produced a draft summary format and applied it to a recent Cochrane review on the effectiveness of portion, package or tableware size for changing selection and consumption of food, alcohol and tobacco [24]. Given rising obesity rates in Europe and much public debate on the best means to tackle this pressing public health problem at national and international levels, including through regulation, this topic was considered to be highly relevant for public health decision-makers in Germany, Austria and Switzerland.

In order to receive feedback early on in the process and to test the interview guide and analytical framework, we performed one pre-test in each of the participating countries (three in total) with representatives of our target audience with whom we had established ties (Fig. 1). Based on the findings from the pre-tests, we revised the draft summary format and developed the prototype summary format (Additional file 2). On the first page, this preliminary version of the summary format contained the review’s title, a short background statement, a box with the review’s key messages and a table listing the review’s inclusion criteria and main characteristics of the studies included. The second and third pages presented the SoF tables and a small box explaining the GRADE rating. The fourth and final page contained a box entitled ‘Relevance for decision-makers’ listing information on differential effects by subgroup, funding and conflicts of interest and relevance for public health practice, as well as a box entitled ‘Further information’ with the bibliographic reference of the systematic review, the authors of the summary and relevant web-links.

### User tests

We performed user tests guided by the ‘think-aloud’ method described in the ‘User Test Package’ of Cochrane Norway [25]. In user tests applying the think-aloud method, representatives of the target audience are asked to continuously verbalise their thoughts as they use a prototype of a product, while researchers observe them and listen to their comments [26]. The aim is to obtain a better understanding of the user experience, to observe the users’ problems with the product, to collect their suggestions and to improve the product on the basis of the structured observations and participant feedback. It has been applied to a variety of Cochrane products [27–29] and other knowledge translation formats [30, 31].

### Participant inclusion criteria and the recruitment process

We set out to recruit at least six participants in each of the three countries (18 in total), representing different institutions and levels of decision-making. For the purposes of this project, we defined public health decision-makers as those responsible for or involved in formulating or financing public health-relevant policies, programmes and regulatory measures. In each country, members of the research team identified relevant institutions representing public health decision-makers at national and sub-national levels (i.e. Bavaria for Germany, German-speaking part of Switzerland, lower Austria for Austria). Within these institutions, we identified departments relevant to our work and with whom we either had existing ties or with whom we were planning to establish such ties in the future. We recruited participants to cover a wide range of decision-making experiences and contexts, including heads of department (strategic decision-making) as well as members of staff with responsibility for specific programmes and content matter expertise (programmatic decision-making). We sent an invitation letter to identified individuals within relevant institutions informing them about the aims and process of the study. In case of no response, reminders were sent after 1–2 weeks or the invited individuals were contacted by phone.

### User test procedure

User testing sessions were conducted face-to-face with individual participants and facilitated by one researcher at each site (LKB, MM, CK). Time and place were agreed upon with each participant individually; user testing sessions took place between June and August 2016, usually at the participant’s workplace. Participants were asked whether their contribution could be recorded and were assured anonymity. Every interviewee agreed to these terms and provided written informed consent. At the start of the interview, the participant was given a printed copy of the prototype summary to read on their own. Using a semi-structured interview guide, the interviewer then led participants through each part of the document, encouraging them to keep up a running monologue. The interview guide (Additional file 3) was based on the framework for user experience by Morville [32], which was adapted by Rosenbaum [33]. The eight facets that we chose to cover in our interview guide were credibility, usability, understandability, usefulness, desirability, identification, value and findability. An additional ninth facet, accessibility, assessed where the participant would expect to find the summary, and was included to help us develop a dissemination strategy and place future systematic review summaries appropriately.

The sessions were audio-taped and the interviewer took notes on the participant’s monologue and on their observations of the user experience. Finally, the interviewers asked the participants additional questions, e.g. about barriers to the use of systematic reviews in their everyday working life and their familiarity with systematic reviews.

### Analysis

At each of the three study sites, one researcher (LKB, MM, CK) excerpted relevant passages from the respective audio recordings. All data were de-identified before analysis. In a first round, each researcher paraphrased the relevant excerpt, entered it into Microsoft Excel, and manually coded it against the nine pre-determined facets, related it to the section of the product concerned (e.g. SoF table, key messages, GRADE, background), and categorised its quality (general comment; low, medium or serious problem; explicitly positive statement). A second researcher (LKB, MM, CK) checked the initial codings; disagreements were resolved within the team to ensure a consistent coding process. The coded excerpts were analysed for themes and issues that commonly applied across countries. The results of this analysis informed the content and design changes to the summary format.

### Revision of the summary format

The findings were summarised according to the section of the summary concerned and the quality of the comment. Within the team, we discussed how the participants’ comments and problems could be addressed; one researcher (JMS) then developed suggestions based on these discussions. Where several options were available, a communication specialist was consulted. The format was thus revised in an iterative process based on several rounds of discussion and revision within the group of researchers. We based the decision on how to handle the participants’ comments primarily on the feasibility of integrating them within the summary format, the pertinence of the comment and the recommendation of the communication specialist.

## Results

### Participants

We conducted 18 interviews, six at each participating study site. Decision-makers were affiliated with different national and regional public health institutions, predominantly government agencies, and represented different levels of seniority ranging from research associate to head of department. Participants had been working in their current position between 6 months and 15 years. When asked whether they believed that the use of research evidence was valued in their position, 16 answered affirmatively, but four of them did not believe that it was common practise. All but one participant said they knew what a systematic review was, 12 said they had read one, and five had read selected chapters or a summary of a systematic review.

In the analysis, we could not discriminate the facet ‘value’ from other facets and found that participants’ responses to that question were already covered in previous responses. We therefore decided not to analyse this facet separately and reported related aspects under the best-fitting facet.

Overall, the summary was well-received and the participants indicated interest in receiving information about systematic reviews presented in this manner. The following sections present findings across the three countries for each of the eight facets.

### Credibility: is the product trustworthy?

At the beginning of the interview, the participants were asked how they rated the document’s credibility based on their first impression. Most considered the summary to be trustworthy. A large majority attributed this to the Cochrane logo placed on top of the first page.

“*If it says Cochrane, I know, I can trust it*.”

Another reason mentioned frequently was the provision of numbers and the inclusion of the grading of the certainty of the evidence.

“*I see RCTs, I see concrete numbers, specifications regarding percentages, participants – it does give the impression that it was carefully researched and it seems that it is a reliable source, that I would trust.*”

Furthermore, participants assumed credibility of the summary because it referred to the conflict of interest statements of the included primary studies and followed a clear and transparent design. Nevertheless, participants missed information that can be expected in a document from a credible source, such as a publication date and the names of the authors of the summary.

### Usability: how easy and satisfying is this product to use?

Many aspects of the layout and structure (e.g. headings and sub-headings, boxes) received praise. The box ‘Relevance for decision-makers’ and key messages were considered to be the most important sections of the summary.

Many participants described the information density as overwhelming and found that it hindered easy use of the document and quick extraction of relevant information. Feedback regarding the SoF table was particularly controversial. On the one hand, participants praised its structure and noted that they generally liked tables to obtain an overview. On the other hand, the actual use of the SoF tables presented a challenge, where several participants said that they preferred text and would like to receive more explanations. Another factor that limited the summary’s usability was the time needed to read and actually understand it.

“*If I want to understand the text in-depth, then I would need 10–15 minutes. That’s long. That’s a lot of time. To read the key messages, which frequently show up in practice, 1–1.5 minutes is the most. If I can’t find what I am looking for, I put it aside*.”

The participants suggested a range of specific changes to formatting and structure that would make the document easier to use, e.g. more prominent sub-headings and placing the box ‘Relevance for public health practice’ together with the key messages on the first page.

### Understandability: do users recognise the product category and understand its content?

Most participants found the text-based parts of the summary understandable and the wording appropriate for decision-makers, public health practitioners and lay people.

However, understanding the SoF tables and GRADE ratings caused considerable problems. In the SoF tables, the most important source of confusion were statistical terms such as standard deviation, confidence interval, relative effect, illustrative comparative risk, outcome, and the differences between studies and independent comparisons. Participants appreciated the interpretation aid and said it helped them understand the tables, but additionally suggested the inclusion of the definitions of statistical terms used.

“*This is quite a lot of information in such limited space, particularly in the* [SoF] *tables. I have the impression that they* [the tables] *are geared towards people with a strong background in statistics. For example: ‘standard deviations higher’. And it is also somehow assumed that one is familiar with the evidence rating.* [...]. *I am not sure that this statistical language is ideal for decision-makers.*”

The explanations provided about GRADE and what the different ratings implied did not help participants interpret the results, often because they were not familiar with the term ‘effect estimate’. It was also mentioned that there was a discrepancy between the graphical representation of the ratings and the labels used to describe them, e.g. three out of four points to indicate ‘moderate’ quality. They also pointed out that ‘moderate’ could be interpreted very differently from its intended meaning and therefore recommended only stating the symbols.

“*From our own studies, we know that people interpret these labels very differently. For me,* [‘moderate’] *would mean ‘in the middle’. This is not consistent with three crosses out of four. Therefore, symbols would be clearer to me*.”

The participants also wanted information about what GRADE actually implies and how authors arrived at the respective rating.

### Usefulness: does this product have practical value for users?

Most participants stated that a summary was useful because it would allow them and their colleagues to obtain a quick overview of the respective topic. Moreover, the language used was considered to be appropriate for both experts and lay people; as the summary was provided by a credible source (Cochrane) it could be presented to external partners such as the Ministry of Health.

“*Yes, very helpful. We have a high workload and we need to get the general idea fast. In the department we mostly have lawyers, who need the results presented in a condensed manner, without too many details*.”

The most cited reasons for limitations in the summary’s usefulness were that the certainty of the evidence (‘moderate’ or lower) did not allow clear conclusions to be drawn and that it did not provide concrete recommendations adapted to the local situation.

“*I would expect to find a recommendation for action, for implementation here*.”

Accordingly, participants suggested giving practical examples of how the results could be implemented. They also said that it would help them if the summary already included contextual information about the legal and political conditions in their respective country or region.

### Desirability: is it something users want and have a positive emotional response to?

Participants much appreciated a summary in German that was easy to understand. The elements carrying a lot of information condensed into simple messages, e.g. key messages, the graphical GRADE ratings and the box on ‘Relevance for decision-makers’, were found particularly appealing.

“*What I thought was good is the box with the key messages. I would, of course, dive into it right away*.”

Many participants disliked the high density of information, particularly in the SoF tables. Graphical illustrations and more explanations in text form were two examples named to overcome these problems.

### Findability: can users locate what they are looking for?

Participants liked the visual emphasis of important information, either by placing such information in a box or by having key terms in bold letters.

“*What I always like is that the key messages, like an abstract, including the results can be easily found right at the beginning, I find that nice because they are also visually highlighted*.”

They also said that some aspects of the formatting made them miss important information, e.g. they overlooked the sub-headings because the font colour was too faint and the surrounding elements very dominant. One participant was confused because the results on alcohol-specific interventions were not found, although this was explicitly described in the title and background as one aspect of the review question.

### Identification (Affiliation): do users feel that the product is for people like them?

Participants’ opinions diverged as to whether the summary was for people in positions like their own. Some welcomed the initiative and said that they were looking forward to receiving more such summaries. Others did not think of themselves as the right audience for this format because they lacked the necessary statistical knowledge or needed more comprehensive information.

“*The question is who should use this* [summary]. […] *If it is for research associates who, at least in theory, have some statistical training, then it is fine exactly the way it is.*”

“*If I were better at statistical things, but that is just my personal background* […] *My background is specifically qualitative* …”

On a similar note, several participants said that the depth of information was too much for political decision-makers. Title, key messages and a link to more detailed results would be more suitable for this audience.

### Accessibility: where would users expect to find the product? How can it be made more accessible?

When asked where they would expect to find systematic review summaries, most participants named those web-based sources where they would usually look for scientific information namely Google, Google Scholar, PubMed, the Cochrane Library, and the homepages of academic and government institutions. When asked how these summaries could be best disseminated, most of them mentioned newsletters and mailing lists and explained that they did not want to have to actively look for them. One participant, however, pointed out that a summary may easily get lost in the daily flood of emails. The need for advertising, for example, at suitable conferences, was emphasised.

### Barriers to the use of evidence from systematic reviews

When asked what stopped them from using evidence from systematic reviews, half of the participants named time constraints. The second most frequent reason was somehow related, i.e. that reviews are usually published in English, which takes longer to read and is more difficult to understand than one’s native language.

“*We all speak English to some degree. But it does make a difference whether I have a review in English in front of me and for only a quick reading think ‘Hmm, not for now.’ But if I have this* [summary] *in German with key messages and* [a section] *‘Relevance for decision-makers’ I think ‘Oh, yes, that does look quite interesting, I will have a quick look.’ That definitely helps*.”

Other reasons mentioned were lacking access to publications and that reading scientific studies did not fall within their area of responsibility but within that of their members of staff.

### Changes to the summary format

We made five substantive changes on the basis of user test results:

1. Flexible length of summaries: As several participants stated that they would rather receive the information on more but better-structured pages, we departed from our initial plan of restricting the summary to four pages to make the document more readable. Depending on the systematic review, the summary may range from four to seven pages.
2. Key messages and relevance for public health practice on front page: As many participants considered the key messages and ‘Relevance for public health practice’ to be the most important components, we moved these to the first page. This should allow readers to quickly scan the document for relevance and then, if desired, assess detailed information on the following pages.
3. Expanded interpretation aid: All participants liked the interpretation aid, but nevertheless had problems understanding the results due to the technical terms used and their limited statistical literacy. We expanded the interpretation aid and added an example on what these results could mean for a real-world setting for each outcome in the tables.
4. Standardised glossary: All participants liked the SoF table, but most had problems understanding the information. We added a glossary on the final pages, in which key technical terms are defined using simple language. This glossary will be included with every summary.
5. Presentation of GRADE ratings: Participants, most of whom were not familiar with the GRADE approach, reported that a GRADE rating of less than ‘high’ would lead them to dismiss the findings as unusable. We therefore removed the attributes describing the certainty of the evidence (e.g. ‘moderate’) and solely display this information using symbols.

With support from a health communication specialist, we made a number of additional minor changes to the structure and layout of the summary format (e.g. colour shadings). We also added the names of the authors of the summary on the last page of the summary format. The revised example summary for the Cochrane review on ‘Portion, package or tableware size for changing selection and consumption of food, alcohol and tobacco’ was translated into English and is available online (Additional file 4). Table 1 provides a comparison of the structure of the prototype and revised summary format.

**Table 1:** Comparison of the structure of prototype and revised summary format

	Prototype Summary Format	Revised Summary Format
Page 1	Title; Background, box with Key messages; table listing the review’s inclusion criteria and characteristics of the studies included	Title; box with Key messages categorised by Background, Results, Scientific Background; box ‘Relevance for Public Health Practice’
Page 2	Results in Summary of Findings (SoF) table; box with explanation of GRADE ratings	Table listing the review’s inclusion criteria and characteristics of the studies included; box with explanation of GRADE ratings
Page 3	Results in SoF table	Results in SoF table
Page 4	Box ‘Relevance for decision-makers’ listing information on ‘Differential effects by subgroup’, ‘Funding and conflicts of interest’, and ‘Relevance for Public Health Practice’, Box "Further Information"	Results in SoF table
Page 5		Boxes listing information on ‘Differential effects in subgroups and special characteristics’, ‘Study funding and conflicts of interest’, ‘Further information’
Pages 6/7		Glossary of technical terms

## Discussion

### Key findings

We developed a German language summary format for systematic reviews of public health interventions in an effort to support the knowledge transfer to public health decision-makers in Germany, Austria and Switzerland. Through user tests, we explored our target group’s experience with this summary. Overall, the summary format was well-received and participants indicated their interest in it; they particularly appreciated the key messages, the section ‘Relevance for public health practice’ and to have information readily available in their own language. However, the user tests also revealed a range of problems. Many of these, for example, participants’ difficulties with understanding statistical terms and their wish for a less information-dense structure, were resolved through our revision of the summary format. Others, however, are more difficult to address, for example, the expressed need for recommendations and contextual information, and are discussed in more detail below.

### Comparison with other studies

Participants originated from different professional backgrounds and positions, and hence expressed diverging needs regarding the length of the summaries and related depth of information, ranging from a one-pager to be read in 2 minutes to a more detailed summary of eight pages or more. Sometimes, even individual participants gave conflicting feedback, at one point indicating that the document should be shorter to cater to their limited time frame, at a later point suggesting that more information should be added. Decision-makers’ preference for short texts and simple messages [4, 5, 14, 31], the seemingly contradictory wish for both brevity and detail [30], and the preference for an accessible structure [34] have also been reported by others. The revised summary format should cater to different types of decision-makers. Most likely strategic decision-makers with very limited time will only scan through the first page whereas programmatic decision-makers with more subject-specific knowledge and a scientific interest will use the full summary.

Participants generally appreciated the inclusion of a SoF table and stated that the provision of GRADE ratings conveyed credibility to the information; however, the problems in relation to the interpretation of GRADE ratings are well-known [31, 35]. Because previous user tests [29, 31] had demonstrated difficulties with understanding SoF tables and because a previous study [36] found that users want to have an explanatory comment, we included an interpretation aid in the prototype summary format and expanded this in the revised summary format. Our user tests corroborated the need for detailed explanations of technical terms found in former studies. There might be a learning effect where participants expect to find the SoF tables and GRADE ratings easier to understand upon repeated exposure [29].

Another challenging observation was the participants’ wish for information outside the scope of a systematic review. Many decision-makers deplored the absence of detailed recommendations and guidance for the implementation of findings, an observation also made in other user testing studies on knowledge translation products [30, 31]. Despite acknowledging the importance of these factors, the time and person-power needed to compile such information will make it difficult to produce locally adapted systematic review summaries in a standardised manner and on an ongoing basis.

### Strengths and limitations

A strength of our study lies in the participation of a broad range of public health decision-makers from distinct institutions and areas of competence in Germany, Austria and Switzerland. It reflects the diverse needs of strategic as well as more programmatic decision-makers at national and regional levels. In employing user testing, we pursued a non-quantitative approach to capturing the users’ experience with the systematic review summary. We only included six participants per country and may not have captured the full range of experiences with the product in relation to characteristics of sex, age, sociocultural or professional backgrounds.

We conducted our user testing with the summary of a single recent Cochrane review [24]. It is therefore possible that reactions to the summary format by some participants were specific to the content of this systematic review (e.g. not finding results for alcohol-specific interventions due to the non-availability of eligible studies). In a similar manner, reviews of other public health interventions may present with additional challenges. Further user testing could also serve to explore to what extent it might be useful to split larger reviews into several summaries (e.g. by splitting the systematic review on portion, package or tableware sizes for changing selection and consumption into three shorter summaries focusing on food, alcohol and tobacco, respectively).

The think-aloud method employed in this study has been successfully applied in several previous studies with similar aims [27, 29–31], and we found it very suitable to this study’s needs. However, user testing creates an artificial situation and thus cannot fully reflect the real-life situation it is intended to shed light on. By conducting the user tests in the participants’ work places, where they would usually access and read a systematic review summary, we have attempted to attenuate this problem.

In addition, participants were likely aware that the interviewers had been involved in the preparation of the summaries, and this may have biased their responses. By continuing to encourage participants to think aloud, to describe their problems with the summary and to make suggestions for improvement, we sought to minimise any social desirability bias. One further strength lies in our analytical rigour. Three researchers independently coded and then cross-checked their colleagues’ work, thereby reducing subjectivity of interpretation.

There is one significant limitation with respect to our overall approach, namely that Cochrane systematic reviews focus on the validity of research findings, and – in line with a ‘knowledge shapes policy model’ [37], where evidence affects decision-making through either short-term direct use or longer term thought enlightenment [38] – efforts to increase the uptake of research findings are primarily concerned with better dissemination and communication of evidence. However, uptake of research findings also requires that evidence obtained through a global systematic review be examined with respect to its applicability in a local decision-making context. A systematic review summary format can help with this translation from generic findings to specific circumstances, but the summary format by itself is not sufficient to enable evidence uptake and use.

## Conclusions

Public-health decision-makers in Germany, Austria and Switzerland welcomed the development of a German language summary format for systematic reviews of public health interventions and indicated their need for and intention to use systematic review summaries presented in this manner. Most participants in the user tests found the prototype summary format useful and a credible source of information, but the user tests also revealed their problems with understanding and interpreting the findings. As much as possible, the participants’ reflections and suggestions were incorporated in the revised summary format. This revised summary format will be used by CPHE to communicate the results of current and future Cochrane reviews of public health interventions. By itself, it will not be sufficient to increase the uptake and use of evidence in decision-making, but must be complemented by larger ‘exchange’ efforts. Feeding back to participants and providing summaries of systematic reviews that respond to their specific information needs presents an important entry-point for establishing longer-term relationships between research and policy and practice, and facilitating exchange efforts to inform evidence-informed decision-making.

Based on the summary format developed for and tested in German-speaking countries, we plan to also develop and test an English version. Depending on the experience with both language versions, we also envisage versions to be developed for other languages. In addition, we will explore whether an online summary format is worthwhile, which could contain both standard sections as well as optional sections with variable content designed for specific target audiences such as decision-makers at different levels or the media. We invite users to share their experience with the summary format – this will help us to further improve the uptake of research evidence in public health decision-making.

## Additional files

Additional file 1: Inventory of existing tools for summarising the findings of systematic reviews in the health field (last search 03/2017). (DOCX 33 kb)

Additional file 2: The prototype summary format applied to the Cochrane review on ‘Portion, package or tableware size for changing selection and consumption of food, alcohol and tobacco’ (in German). (PDF 126 kb)

Additional file 3: The interview guide as used in the user testing (in German). (DOCX 48 kb)

Additional file 4: The revised summary format applied to the Cochrane review on ‘Portion, package or tableware size for changing selection and consumption of food, alcohol and tobacco’ (in English). (DOCX 122 kb)

## Abbreviations

SoF: summary of findings
CPHE: Cochrane Public Health Europe
GRADE: Grading of Recommendations Assessment, Development and Evaluation
