# Supplementary material for: Facilitating evidence uptake: development and user testing of a systematic review summary format to inform public health decision-making in German-speaking countries
Source: Health Res Policy Syst. 2018 Jul 9;16:59. doi: 10.1186/s12961-018-0307-z (PMC6038322; doi:10.1186/s12961-018-0307-z)
Supplement: Supplementary file 1 — Inventory of existing tools for summarising the findings of systematic reviews in the health field (last search 03/2017). (DOCX 33 kb) [file 12961_2018_307_MOESM1_ESM.docx]

**Table S1: Inventory of existing tools for summarising the findings of systematic reviews in the health field**

| Author (Hyperlink: source | Title | Description  (scope, target audience, thematic focus) | Structure/elements | Presentation and access | Length | Tested/ evaluated |
| --- | --- | --- | --- | --- | --- | --- |
|  |  |  | **Summaries of Cochrane reviews** |  |  |  |
| [Cochrane](http://www.cochrane.org/evidence) review authors | Plain language summaries | Stand-alone summary of Cochrane Systematic Reviews written in a straightforward style that can be understood by consumers of health care | [Mandatory Elements](http://editorial-unit.cochrane.org/sites/editorial-unit.cochrane.org/files/uploads/PLEACS_0.pdf):   - Title - Review Question - Background - Search Date - Study Characteristics - Key Results for all Main Outcomes and Adverse Events - Quality of the Evidence (GRADE) | Text only  Online, free availability | Desirable: 400 words, and no more than 700 words | Glenton C, Santesso N, Rosenbaum S, Nilsen E, Rader T, Ciapponi A and Dilkes H [1], Santesso N, Rader T, Nilsen ES, Glenton C, Rosenbaum S, Ciapponi A, Moja L, Pardo JP, Zhou Q and Schünemann HJ [2] |
| [Cochrane Primary Health Care Field](http://www.cochraneprimarycare.org/pearls-and-pearls-various-languages) | Cochrane PEARLS (Practical Evidence of Real Life Situations) | Summaries of Cochrane SR for primary care practitioners. PEARLS provide guidance on the effectiveness of a treatment. | - Title - Clinical Question - Bottom Line - Caveat - Context - Citation - PEARLS author | Predefined structure  Online, free availability | 1 page | Not known |
| [Health Knowledge Network La Trobe University](http://www.latrobe.edu.au/chcp/evidence-bulletins) | Evidence Bulletin | Accessible summaries of Cochrane SR about communicating with and involving consumers and carers for health communication professionals | - Title - Review Question - Explanation of Intervention - Key Findings - Detailed Review Information (Background, Information about this Review, Main Results, Results Table, Limitations, Applicability and Implications for Decision-Makers) - Related Resources | Predefined structure  Online, free availability | 6 pages | Not known |
| [Policy Liaison Initiative](http://policymakers.evidencemap.org/) | CRISP (Cochrane Reviews Identified and Summarised for Policy) | Summaries of Cochrane Reviews focussing on public health, effective practice and organisation of care, health priority consumers and communication topics to foster the use of research for informing policy work | - Title - Important Dates: published, search last updated - Key Messages - Review at a Glance (PICOS, Quality of Included Studies) - Plain Language Summary - Results: SoF-Table - AMSTAR Rating - Online version with download possibilities for review citation, full review, study citations | Predefined structure  Online, free availability | NA | Evaluation of the whole initiative, not specifically the summary format in Brennan et al., 2016 |
|  |  |  | **Not Cochrane-specific summaries** |  |  |  |
| [Centre for Reviews and Dissemination](http://www.crd.york.ac.uk/CRDWeb/), DARE database | Abstracts of Reviews of Effects (DARE) | Quality-appraised summary of single non-Cochrane SR of effects of healthcare or health system interventions (delivery and organization of health services) | - Summary (2-3 sentences) - Author’s Objectives - Searching - Study Selection - Assessment of Study Quality - Data Extraction - Methods of Synthesis - Results of the Review - Author’s Conclusions - Commentary - Implications of the Review for Practice and Research | Text only, predefined structure  Online, free availability | Appr. 2-3 pages | Not known |
| [McMaster University](http://healthevidence.org/search.aspx) | Health Evidence ^TM^ | Summary statements synthesizing results of SR of health promotion and public health interventions for practitioners, decision-makers and managers | - Review Focus (PICO) - Review Quality Rating - Considerations for Public Health Practice - Evidence and Implications - Why this is of Interest to Public Health in Canada | Predefined structure  Online, free availability | 2 pages | Not known |
| [SUPPORT Collaboration](http://www.supportsummaries.org/) | SUPPORT | Structured summaries of SR of effects of healthcare or health systems interventions in maternal and child health in low- and middle income countries | - Key background information - Search Strategy and Results - Main Findings of the Review (QoE) - Relevance of the Review to Low and Middle-Income Countries - Related Resources | Predefined structure  Online, free availability | 5-7 pages | Rosenbaum S, Glenton C, Wiysonge C, Abalos E, Mignini L, Young T, Althabe F, Ciapponi A, Marti S, Meng Q, Wang J, la Hoz Bradford A, Kiwanuka S, Rutebemberwa E, Pariyo G, Flottorp S and Oxman A [3] |
| [The Campbell Collaboration](http://www.campbellcollaboration.org/plain-language-summaries/explore/pls-page) | Plain Language Summary | Summaries of Campbell SR (also non-health related) approved by the lead author for policy makers, practitioners and users | - Background - Review Question - Included Studies - Results of the SR - Interpretation of the Results - Characteristics of the Review in a Box | Text only and pictures  Online, free availability | 2 pages | Not known |
| [The Campbell Collaboration](http://www.campbellcollaboration.org/plain-language-summaries/explore/pls-page) | Policy Briefs | Overview of > 1 Campbell SR (also non-health related) for policy makers | - Background - Included Studies - Results - Implications for Practice - Future Work | Text only and pictures  Online, free availability | 4 pages | Not known |

^1^The formats were identified through the lists provided in Opiyo et al. [4, supplementary table S1], Lavis JN [5] and research done by Turner et al. (Tari Turner, personal communication).Information updated as of 3/2017

Abbreviations: GRADE, Grading of Recommendations Assessment, Development and Evaluation; PICOS, Population, Interventions, Comparisons, Outcomes, Setting; QoE, Quality of Evidence; SoF, Summary of Findings; SR, systematic review

Literature:

- Haynes et al. (1990): <http://annals.org/article.aspx?articleid=703961>
- Petkovic (2016): <https://www.ncbi.nlm.nih.gov/pubmed/27938409>

Criteria for evidence summaries, discussed in the table:

- Evidence summaries for systematic reviews
- Regular publication of evidence summaries (twice a year in the last 2 years, since 2015)
- Public availability
- Public Health relevance

# References

1. Glenton C, Santesso N, Rosenbaum S, Nilsen E, Rader T, Ciapponi A, Dilkes H: **Presenting the results of Cochrane Systematic Reviews to a consumer audience: a qualitative study.** *Med Decis Making* 2010, **30:**566 - 577.

2. Santesso N, Rader T, Nilsen ES, Glenton C, Rosenbaum S, Ciapponi A, Moja L, Pardo JP, Zhou Q, Schünemann HJ: **A summary to communicate evidence from systematic reviews to the public improved understanding and accessibility of information: a randomized controlled trial.** *Journal of Clinical Epidemiology* 2015.

3. Rosenbaum S, Glenton C, Wiysonge C, Abalos E, Mignini L, Young T, Althabe F, Ciapponi A, Marti S, Meng Q, et al: **Evidence summaries tailored to health policy-makers in low- and middle-income countries.** *Bull World Health Organ* 2011, **89:**54 - 61.

4. Opiyo N, Shepperd S, Musila N, Allen E, Nyamai R, Fretheim A, English M: **Comparison of alternative evidence summary and presentation formats in clinical guideline development: a mixed-method study.** *PLoS One* 2013, **8:**e55067.

5. Lavis JN: **How can we support the use of systematic reviews in policymaking?** *PLoS Med* 2009, **6:**e1000141.
