# Supplementary material for: Facilitating evidence uptake: development and user testing of a systematic review summary format to inform public health decision-making in German-speaking countries
Source: Health Res Policy Syst. 2018 Jul 9;16:59. doi: 10.1186/s12961-018-0307-z (PMC6038322; doi:10.1186/s12961-018-0307-z)
Supplement: Supplementary file 2 — The prototype summary format applied to the Cochrane review on ‘Portion, package or tableware size for changing selection and consumption of food, alcohol and tobacco’ (in German). (PDF 126 kb) [file 12961_2018_307_MOESM2_ESM.pdf]

Wirken sich Größe von Portionen, Packungen und Geschirr auf Auswahl und Konsum von Lebensmitteln, Alkohol oder Tabakwaren aus?

Überernährung und der übermäßige Konsum von Alkohol und Tabak stehen in Zusammenhang mit der Entstehung diverser chronischer Krankheiten. Der Bevölkerung werden in Läden, Automaten, Restaurants oder Bars Lebensmittel, Alkohol und Tabakwaren auf verschiedene Art und Weise dargeboten. Für die öffentliche Gesundheit ist es deshalb wichtig zu erfahren, ob sich die **Größe oder Präsentation dieser Produkte** für Interventionen zur Änderung des Konsumverhaltens nutzen lassen.

Kernaussagen

- → Teilnehmer konsumierten durchweg mehr Lebensmittel und alkoholfreie Getränke, wenn ihnen größere Portionen und Packungen oder bestimmte Formen von Geschirr angeboten wurden.
- → Überträgt man diesen Effekt auf die zugeführten Kalorien, entspräche dies bei Erwachsenen in Großbritannien einer höheren Kalorienzufuhr von 12 bis 16 Prozent.
- → Politiken und Praktiken, die die Verfügbarkeit und Attraktivität größerer Portionen und Packungen sowie bestimmter Formen von Geschirr erfolgreich einschränken, können zu einer gesundheitlich relevanten Reduzierung der konsumierten Menge an Essen und nicht-alkoholischen Getränken beitragen.
- → Aufgrund der unzureichenden Datenlage ist es nicht möglich, eindeutige Schlussfolgerungen für Politiken und Praktiken in Bezug auf Tabak und Alkohol zu treffen.

Was der systematische Review untersucht

	**Einschlusskriterien**	**Charakteristika der eingeschlossenen Studien**
**Interventionen**	Veränderungen in Größe und Präsentation von Lebensmitteln, alkoholischen Getränken und Tabakwaren<br><br>Studiendesign: Randomisierte kontrollierte Studien (RCTs)	Insgesamt 72 Studien:<br>• 69 Lebensmittel<br>• 3 Zigaretten<br>• 0 alkoholische Getränke<br>Davon:<br>• 38 Portionsgröße<br>• 12 Packungsgröße<br>• 6 Größe einzelner Produkteinheiten<br>• 16 Größe oder Form von Geschirr
**Teilnehmer**	Kinder und Erwachsene	Insgesamt 6603 Teilnehmer<br>• 5182 Erwachsene in 55 Studien<br>• 1421 Kinder in 17 Studien
**Endpunkte**	Auswahl (selbstgewählte Menge) und Konsum von Lebensmitteln, Alkohol und Tabakwaren	Auswahl 13<br>Konsum 65
**Ort/Kontext**	keine Einschränkung	Studien ausschließlich aus Ländern mit hohem Einkommen, davon 58 in den USA, keine aus D/AT/CH<br>• 50 unter Laborbedingungen<br>• 22 unter realen Bedingungen (Restaurants, Schulen, Kantinen)
**Aktualität:** Juli 2013 (Veröffentlichungsdatum der letzten eingeschlossenen Studie)		

Ergebnisse

1. Wirkung der Größe auf Auswahl und Konsum

Lebensmittel: Größere versus kleinere Portionen, Packungen oder Geschirr

Intervention: Größere Portionen, Packungen oder Geschirr
Teilnehmer: Kinder und Erwachsene
Ort/Kontext: Länder mit hohem Einkommen; Laborbedingungen und reale Bedingungen
Kontrolle: Kleinere Portionen, Packungen oder Geschirr

**Endpunkte**	**Illustrative Risikovergleiche**[1]		**Relativer Effekt (95% Konfidenzintervall)**	**Anzahl der Teilnehmer (unabhängige Vergleiche)**	**Qualität der Evidenz (GRADE, s. Kasten)**
	**Kontrolle: Kleinere Größen**	**Intervention: Größere Größen**			
Auswahl ohne Kauf	1689 kcal[1]	209 (119 bis 293) kcal oder 12.4% mehr als Kontrolle	0.42 (0.24 bis 0.59) Standardabweichungen höher	1164 (13 Vergleiche)	⊕⊕⊕◯ moderat
Interpretationshilfe: Teilnehmer, denen Essen und Trinken in größeren Portionen, Packungen oder Geschirr angeboten wurden, nahmen sich mehr als Teilnehmer denen kleinere angeboten wurden.					
Auswahl ohne Kauf bei Kindern	1651 kcal[1]	63 (27bis 153) kcal oder 3.8% mehr als Kontrolle	0.14 (0.06 bis 0.34) Standardabweichungen höher	382 (4 Vergleiche)	⊕⊕⊕◯ moderat
Auswahl ohne Kauf bei Erwachsenen	1727 kcal[1]	188 (188 bis 403) kcal oder 10.9% mehr als Kontrolle	0.55 (0.35 bis 0.75) Standardabweichungen höher	782 (9 Vergleiche)	⊕⊕⊕◯ moderat
Konsum	1689 kcal[1]	189 (144 bis 228) kcal oder 11.2% mehr als Kontrolle	0.38 (0.29 bis 0.46) Standardabweichungen höher	6603 (86 Vergleiche)	⊕⊕⊕◯ moderat
Konsum bei Kindern	1651 kcal[1]	95 (45 bis 140) kcal oder 5.7% mehr als Kontrolle	0.21 (0.1 bis 0.31) Standardabweichungen höher	1421 (22 Vergleiche)	⊕⊕⊕◯ moderat
Konsum bei Erwachsenen	1727 kcal[1]	247 (215 bis 279) kcal oder 14.3% mehr als Kontrolle	0.46 (0.40 bis 0.52) Standardabweichungen höher	5182 (64 Vergleiche)	⊕⊕⊕◯ moderat

[1] Die angenommenen Werte der Kontrollen beruhen auf Daten repräsentativer Umfragen in Großbritannien (Mittelwerte und Standardabweichungen der täglichen Energiezufuhr bei Kindern und/oder Erwachsenen).

Über die Qualität der Evidenz (GRADE)

⊕⊕⊕⊕ **Hoch:** Wir sind sehr sicher, dass der wahre Effekt nahe bei dem Effektschätzer liegt.

⊕⊕⊕◯ **Moderat:** Wir haben mäßig viel Vertrauen in den Effektschätzer: Der wahre Effekt ist wahrscheinlich nahe bei dem Effektschätzer, aber es besteht die Möglichkeit, dass er relevant verschieden ist.

⊕⊕◯◯ **Niedrig:** Unser Vertrauen in den Effektschätzer ist begrenzt: Der wahre Effekt kann durchaus relevant verschieden vom Effektschätzer sein.

⊕◯◯◯ **Sehr niedrig:** Wir haben nur sehr wenig Vertrauen in den Effektschätzer: Der wahre Effekt ist wahrscheinlich relevant verschieden vom Effektschätzer.

Tabakwaren: Längere versus kürzere Zigaretten

Intervention: Längere Zigaretten
Teilnehmer: Erwachsene[1]
Ort/Kontext: Länder mit hohem Einkommen; Laborbedingungen
Kontrolle: Kürzere Zigaretten

Endpunkte	Illustrativer Risikovergleich[2]		Relativer Effekt (95% Konfidenzintervall)	Anzahl der Teilnehmer (unabhängige Vergleiche)	Qualität der Evidenz (GRADE, s. Kasten)
	Kontrolle: Kürzere Zigaretten	Intervention: Längere Zigaretten			
Konsum	13 Zigaretten[2]	2 (1 bis 5) Zigaretten mehr als Kontrolle	0.25 (0.14 bis 0.65) Standardabweichungen höher	108 (6 Vergleiche)	⊕⊕◯◯ niedrig

Interpretationshilfe: Teilnehmer, die längere Zigaretten erhielten, rauchten insgesamt und umgerechnet auf ein Standardzigarettenmaß mehr als Raucher, die kürzere Zigaretten erhielten.

[1] Für Kinder ist keine Evidenz vorhanden.
[2] Die angenommenen Werte der Kontrollen beruhen auf Daten einer repräsentativen Umfrage in Großbritannien (Mittelwerte und Standardabweichungen der gerauchten Zigaretten).

2. Wirkung der Form auf Auswahl und Konsum

Nicht-alkoholische Getränke (Wasser und Fruchtsäfte): Kürzere, breitere versus höhere, schmälere Gläser oder Flaschen

Intervention: kürzere und breitere Gläser oder Flaschen (bei identischem Volumen)
Teilnehmer: Erwachsene und Kinder
Ort/Kontext: Länder mit hohem Einkommen; reale Bedingungen
Kontrollgruppe: höhere und schmälere Gläser oder Flaschen

Endpunkte	Illustrativer Risikovergleich[1]		Relativer Effekt (95% Konfidenzintervall)	Anzahl der Teilnehmer (unabhängige Vergleiche)	Qualität der Evidenz (GRADE, s. Kasten)
	Kontrolle: Kürzer, breiter	Intervention: Höher, schmäler			
Auswahl ohne Konsum	234 g Getränk[1]	242 (86 bis 400) g oder 103.4% mehr als Kontrolle	1.47 (0.52 bis 2.43) Standardabweichungen höher	232 (3 Vergleiche)	⊕⊕◯◯ niedrig
Interpretationshilfe: Teilnehmer, die ein Getränk in höheren, schmäleren Gläsern oder Flaschen einmalig angeboten bekamen, schenkten sich mehr ein als Teilnehmer, die ein Getränk in kürzeren, breiteren bekamen. Das Volumen der Gefäße war in den Gruppen identisch.					
Auswahl ohne Konsum bei Kindern	228 g Getränk[1]	377 (292 bis 462) g oder 165.5% mehr als Kontrolle	2.31 (1.79 bis 2.83) Standardabweichungen höher	96 (1 Vergleich)	⊕⊕◯◯ niedrig
Auswahl ohne Konsum bei Erwachsenen	245 g Getränk[1]	171 (68 bis 274) g oder 70.1% mehr als Kontrolle	1.03 (0.41 bis 1.65) Standardabweichungen höher	136 (2 Vergleiche)	⊕⊕◯◯ niedrig
Konsum bei Erwachsenen[2]	245 g Getränk[1]	195 (95 bis 296) g oder 79.6% mehr als Kontrolle	1.17 (0.57 bis 1.78) Standardabweichungen höher	50 (1 Vergleich)	⊕◯◯◯ Sehr niedrig

[1] Die angenommenen Werte der Kontrollen beruhen auf Daten repräsentativer Umfragen in Großbritannien (Mittelwerte und Standardabweichungen einer Portion eines kalorienhaltigen, nicht-alkoholischen Getränks bei Kindern und/oder Erwachsenen).
[2] Für Kinder ist keine Evidenz vorhanden.

Für Entscheidungsträger relevante Aspekte

Unterschiedliche Effekte in Subgruppen
**Alter**: Die Wirkung auf den Konsum von Lebensmitteln ist altersabhängig mit einem größeren Effekt bei Erwachsenen (92 unabhängige Vergleiche). Die Wirkung des Alters auf die Auswahl von Lebensmitteln konnte aufgrund der unzureichenden Datenlage nicht untersucht werden.<br>**Geschlecht**: In Meta-Analysen konnten keine Unterschiede nachgewiesen werden.<br>**Sozioökonomischer Status**: Konnte aufgrund der unzureichenden Datenlage nicht untersucht werden.<br>**Andere Faktoren**:<br>• Lebensmittel: Die Effekte hinsichtlich des Konsums waren größer in Studien mit weniger gesunden oder kalorienreicheren Lebensmitteln.<br>• Body-Mass-Index: Die Wirkung des Body-Mass-Indexes auf Auswahl und Konsum konnte aufgrund der unzureichenden Datenlage nicht untersucht werden.
**Studienfinanzierung und Interessenskonflikte**
In allen Studien wurden die Finanzierungsquellen offengelegt, und es gab keine Hinweise auf potenzielle Interessenskonflikte.
**Praxisrelevanz**
Politiken und Praktiken, die die Verfügbarkeit kleinerer Portionen, Packungen oder bestimmter Formen von Geschirr fördern, können zu einer gesundheitlich relevanten Reduzierung des Konsums von ungesundem Essen und (nicht-alkoholischen) Getränken beitragen. Dies kann beispielsweise durch zweitrangiges Platzieren größerer Packungen, vorrangigen Angeboten von kleineren Portionen in Restaurants oder Läden oder durch ökonomische Anreize erreicht werden.

Weiterführende Informationen

Der systematische Review, auf dem diese Zusammenfassung beruht
Hollands GJ, Shemilt I, Marteau TM, Jebb SA, Lewis HB, Wei Y, Higgins JPT, Ogilvie D. Portion, package or tableware size for changing selection and consumption of food, alcohol and tobacco. Cochrane Database of Systematic Reviews 2015, Issue 9. Art. No.: CD011045. DOI: 10.1002/14651858.CD011045.pub2 (http://bit.ly/1Rw43eJ)

Cochrane Glossar: http://www.cochrane.de/de/cochrane-glossar
Cochrane Public Health: http://ph.cochrane.org/
Cochrane Public Health Europe: http://ph.cochrane.org/cochrane-public-health-europe

Kontakt
Cochrane Public Health Europe: cochranepublichealth@ibe.med.uni-muenchen.de
Deutschland: Eva Rehfuess, Ludwig-Maximilians-Universität München, rehfuess@ibe.med.uni-muenchen.de
Österreich: Christina Kien, Donau-Universität Krems, christina.kien@donau-uni.ac.at
Schweiz: Margot Mütsch, Universität Zürich, margot.muetsch@uzh.ch, Aline Flatz, Universitätsspital Lausanne und Universität Lausanne, aline.flatz@chuv.ch
