# Supplementary material for: Facilitating evidence uptake: development and user testing of a systematic review summary format to inform public health decision-making in German-speaking countries
Source: Health Res Policy Syst. 2018 Jul 9;16:59. doi: 10.1186/s12961-018-0307-z (PMC6038322; doi:10.1186/s12961-018-0307-z)
Supplement: Supplementary file 3 — The interview guide as used in the user testing (in German). (DOCX 48 kb) [file 12961_2018_307_MOESM3_ESM.docx]

CPHE Summary Format User Testing Interviewleitfaden

| **ModeratorIn** | Name | Vorname |
| --- | --- | --- |
|  | Institution | |
| **BeobachterIn**  **(falls anwesend)** | Name | Vorname |
|  | Institution | |
| **TeilnehmerIn** | Name | Vorname |
|  | Institution | |
|  | Position | |
| **Ort** | Stadt | Land |
| **Datum (tt/mm/jj)** |  | |
| **Titel des syst. Reviews** |  | |

1. **Checkliste**

für ModeratorIn:

- 2 Zusammenfassungen, eine für den Teilnehmer, eine um Anmerkungen zu machen
- Tonbandgerät
- Ersatzbatterien
- Teilnehmerinformation und Einverständniserklärung
- Notizblock

für BeobachterIn (Falls Anwesend)

- Zusammenfassung
- Notizblock

1. **Einleitung**

**Was wir testen und warum?**

Wir werden die Benutzerfreundlichkeit eines von Cochrane Public Health Europe entwickelten Formats testen, welches die Ergebnisse eines systematischen Reviews im Bereich Public Health zusammenfasst.

CPHE ist ein Ableger der in Melbourne, Australien, beheimateten Cochrane Public Health Review Gruppe, einer der insgesamt über 50 themenbezogenen Arbeitsgruppen des internationalen Forschungsnetzwerkes Cochrane.

Sie sind eine von mehreren Personen aus Deutschland, Österreich und der Schweiz, von denen wir Feedback zu diesem Format bekommen möchten. Wir werden dieses Feedback nutzen, um unsere Formate zu verbessern und sie so benutzerfreundlich wie möglich für Personen wie Sie zu gestalten.

**Was werden wir tun?**

Zunächst werde ich Ihnen einige Fragen zu Ihrem Hintergrund stellen.

Dann werde ich Ihnen das Format zeigen und Ihnen Fragen dazu stellen.

Wir möchten dass Sie diese aus Ihrer eigenen Perspektive heraus beantworten und nicht im Namen anderer.

Wenn Sie zustimmen, wird diese Sitzung auf Band aufgenommen.

Die Aufnahmen werden auf relevante Passagen hin analysiert und partiell transkribiert. Die Zwischenergebnisse werden **anonymisiert** an das leitende Institut (LMU München) gesendet.

Die Tonbandaufzeichnungen bleiben in den durchführenden Instituten und werden nach dem zweiten Treffen gelöscht.

Die Sitzung wird insgesamt etwa eine Stunde dauern.

**Über User Testing**

Unserer Erfahrung nach ist es so, dass die Dinge, die Sie schwierig zu verstehen finden auch andere Leute schwierig finden. Daher können wir diese Informationen nutzen, um unser Format zu verbessern. Wir möchten herausfinden, was gut oder weniger gut funktioniert. Dabei geht es uns insbesondere um

- Inhalt
- Sprache und Terminologie
- Darstellung und Formatierung.
- Wir möchten vor allem IHRE EIGENE MEINUNG hören. Daher gibt es keine richtige oder falsche Antwort auf unsere Fragen. Wir testen nicht Sie sondern unser Format.

**Wer macht was?**

Ich werde durch den Test leiten und (*Name des/der BeobachterIn, falls anwesend,* wird) Notizen machen.

Haben Sie irgendwelche Fragen zu dem Projekt?

- **Lasse den/die TeilnehmerIn die Einverständniserklärung unterschreiben**
- **Schalte das Tonbandgerät ein.**

1. **Hintergrundinformationen – 5 Minuten**

- **Lasse den Teilnehmer wissen, dass wir nur etwa 5 Minuten auf die Hintergrundfragen verwenden werden**

| **A** | Haben Sie einen Abschluss in einer (oder mehreren) dieser Fachrichtungen? |
| --- | --- |
| Medizin  Ja  Nein  Naturwissenschaften  Ja  Nein  Gesundheitswissenschaften oder Public Health  Ja  Nein  Sozialer Beruf  Ja  Nein  Gesundheitsberuf (Pflege, Physiotherapie, etc.)  Ja  Nein  Psychologie  Ja  Nein  Anderer: | |

| **B 1** | In welcher Position sind Sie derzeit tätig? | |
| --- | --- | --- |
|  | | |
| **B 2** | | Wie lange sind Sie schon in dieser Position tätig? |
|  | | |

| **C** | Lesen Sie manchmal wissenschaftliche Studien im Zusammenhang mit Ihrer Arbeit und wenn ja, wie häufig? |
| --- | --- |
|  | Nie  < 1x/Monat  1-3x/Monat  1x/Woche  mehrmals pro Woche |

| **D** | Denken Sie bitte an ein Beispiel für eine politische oder programmatische Entscheidung, an der Sie vor kurzem beteiligt waren – welche Art von Informationen (wissenschaftliche Artikel, Berichte, Richtlinien, Meinung von Experten) haben Sie verwendet und wo haben Sie nach diesen Informationen gesucht? (Internetseiten, wissenschaftliche Zeitschriften/Journals, Kollegen, etc.) |
| --- | --- |
|  | |

| **E** | Glauben Sie, dass in Ihrer Position die Verwendung von Evidenz aus Studienresultaten  a) verbreitet ist  b) geschätzt wird | **Ja  Nein**  **Ja  Nein** |
| --- | --- | --- |

Erfahrungen mit systematischen Reviews

| **F** | Haben Sie bereits von systematischen Reviews gehört? | **Ja  Nein** |
| --- | --- | --- |

| **G** | Wissen Sie was systematische Reviews sind? | **Ja  Nein** |
| --- | --- | --- |

| **H** | Haben Sie schon einmal einen systematischen Review, Auszüge daraus oder eine Zusammenfassung davon gelesen? |
| --- | --- |
|  | Einen ganzen oder den Großteil eines systematischen Reviews  Auszüge (einzelne Kapitel)  Eine Zusammenfassung  Nein |

| **I** | Haben Sie schon einmal von Cochrane Reviews gehört? | **Ja  Nein** |
| --- | --- | --- |

| **J** | Wie vertraut sind sie mit Cochrane Reviews? |
| --- | --- |
| nicht vertraut  ich lese/überfliege sie nur selten  ich lese/überfliege sie hin und wieder  ich lese/überfliege sie regelmäßig  ich bin AutorIn oder Co-AutorIn eines Cochrane Reviews | |

**Wiederhole die Richtlinien für das User Testing**

Eine kurze Wiederholung bevor wir anfangen

- **Es gibt keine richtigen oder falschen Antworten**

Wir wollen nicht Sie, sondern unsere Zusammenfassung testen. Es gibt keine richtigen oder falschen Antworten auf unsere Fragen. Wenn Sie finden dass etwas einfach oder schwierig, klar oder verwirrend ist und wenn sie etwas verstehen oder auch nicht, dann möchten wir das gerne wissen.

- **Denken Sie laut**

Denken Sie laut. Sagen Sie mir was Sie denken, was Sie sehen, das Sie auch nur ein wenig verwirrend oder überraschend finden.

Zum Beispiel:

Beschreiben Sie Ihre Erfahrung mit der Zusammenfassung, die wir Ihnen gegeben haben.

Wenn Sie sich bei irgendetwas unsicher sind

Wenn Sie irgendetwas überrascht

Wenn es etwas gibt, das Sie nicht verstehen, sagen Sie einfach “Ich verstehe nicht was das heißen soll…”

- **Meine Rolle**

Meine Rolle hierbei ist es Fragen zu stellen. Da wir in erster Linie an Ihrer Meinung interessiert sind werde ich ansonsten möglichst wenig sagen. Sie können mir Fragen stellen, aber ich werde sie wahrscheinlich nicht beantworten. Falls Sie möchten kann ich sie beantworten wenn wir fertig sind.

**Das Dokument**

- **Warte bevor du die Zusammenfassung zeigst. Lese zuerst den ersten Teil von Frage 1.**

Erste Eindrücke

| **1** | - **Bevor die Zusammenfassung gezeigt wird**   Ich werde Ihnen nun die Zusammenfassung eines systematischen Reviews zeigen. Sie ist ein Beispiel für eine Reihe von Zusammenfassungen, die standardmäßig für Cochrane Public Health Reviews entwickelt werden soll. Ich möchte, dass Sie sich vorstellen, dass Sie diese Zusammenfassung als einen Link auf einer Webseite gefunden haben, die Sie oft besuchen, und dass Sie sich dazu entschlossen haben, diesen Link anzuklicken.  Wenn ich Ihnen die Zusammenfassung gebe, möchte ich Ihren ersten, unmittelbaren Eindruck, Ihre spontane Reaktion. Denken Sie nicht nach, sagen Sie mir einfach das erste was Ihnen einfällt.   - **Gib Ihm/Ihr die Zusammenfassung**   Was ist Ihre erste spontane Reaktion? |
| --- | --- |
|  | |

Wie würden sie normalerweise so eine zusammenfassung lesen?

| **2** | Zeigen Sie mir bitte wie Sie normalerweise beim Lesen einer solchen Zusammenfassung vorgehen würden. Wo würden Sie beginnen, wonach würden Sie zuerst suchen, welchen Teil würden Sie lesen? Wieviel Zeit würden Sie sich üblicherweise nehmen, eine solche Zusammenfassung komplett durchzulesen? |
| --- | --- |
|  | |

Glaubwürdigkeit

| **3** | Basierend auf diesem ersten Eindruck, können Sie uns sagen wie Sie seine Glaubwürdigkeit einschätzen? Glauben Sie dass Sie dieser Information trauen würden? Was macht für Sie eine Information glaubwürdig?   - **Frage ggf. noch einmal gezielt nach (probing):**   Autoren oder Institutionen, die die Zusammenfassung verfasst haben; Herausgeber; Aktualität der Informationen; Transparenz der Quellen; Aufmachung des Dokuments |
| --- | --- |
|  | |

**Sage: Wir werden Ihnen jetzt Zeit geben die Zusammenfassung zu lesen. Sie dürfen sich dafür so viel Zeit nehmen, wie sie möchten.**

- **Erinnere sie daran, dass du danach keine prüfungsähnlichen Fragen stellen wirst.**
- **Verlasse das Zimmer während sie die Zusammenfassung alleine lesen.**
- **Falls der/die TeilnehmerIn dies wünscht kannst Du ihm/ihr die Fragen auch vorlegen, damit er/sie mitlesen kann.**

**Nach dem Lesen der zusammenfassung**

Verwendbarkeit & Benutzerfreundlichkeit, Auffindbarkeit von Informationen

| **4** | Ich möchte, dass Sie nun jedes Element der Zusammenfassung durchgehen, und mir beschreiben wie Sie dieses verstehen.  Bitte beginnen Sie hier oben auf der ersten Seite. Erzählen Sie mir einfach, ob Ihnen Dinge klar oder unklar sind, oder ob es irgendetwas gibt das fehlt und wonach Sie suchen würden. |
| --- | --- |

| **4a** | Titelseite: Titel, Logos |
| --- | --- |
|  | |
| **4b** | Titelseite: Einführendes Statement/kurzer Hintergrund |
|  | |
| **4c** | Titelseite: Kernaussagen |
|  | |
| **4d** | Titelseite: Was der systematische Review untersucht |
|  | |
| **4e** | Seiten 2&3: Summary of Findings Tabellen   - **Gehe jedes Element der Tabellen durch** - **Frage den/die TeilnehmerIn wie er/sie das Ergebnis interpretiert und bitte ihn/sie, das Ergebnis in eigenen Worten wiederzugeben** |
|  | |
| **4f** | Seite 4: Public Health-relevante Aspekte |
|  | |
| **4g** | Seite 4: Weiterführende Informationen |
|  | |

VERSTÄNDLICHkeit

| **5** | Glauben Sie die Zusammenfassung war im Allgemeinen eher leicht oder schwierig zu verstehen? Erklären Sie bitte warum (nicht) … |
| --- | --- |
|  | |

Praktischer Nutzen

| **6a** | Wäre diese Zusammenfassung nützlich für Sie, wenn Sie eine Entscheidung treffen müssten?  Wenn nicht, wie sollte eine solche Zusammenfassung aussehen, damit sie für eine Entscheidung nützlich ist?   - **Frage noch einmal gezielt nach, falls der/die TeilnehmerIn die Frage nicht versteht.**   Was würden Sie ändern? Was fehlt Ihnen? |
| --- | --- |
|  | |
| **6b** | Könnten Sie eine Entscheidung anhand dieser Zusammenfassung treffen oder bräuchten Sie zusätzliche Informationen? Wenn ja, welche zusätzliche Informationen würden Sie benötigen? Wo würden Sie diese suchen? |
|  | |

WÜNSCHENSWERT

| **7** | Gefiel Ihnen die Zusammenfassung (insofern man von „gefallen” sprechen kann)? Würden Sie Informationen gerne auf diese Weise aufbereitet erhalten? Erklären Sie bitte warum (nicht)?   - **Frage gegebenenfalls noch einmal gezielt nach**   Was halten Sie vom Inhalt, Sprache und Terminologie, Darstellung und Formatierung? |
| --- | --- |
|  | |

Mehrwert und Identifikation

| **8** | Glauben Sie solche Zusammenfassungen wären nützlich und wichtig für Ihren Berufsalltag und für Personen in ähnlichen beruflichen Positionen? |
| --- | --- |
|  | |

Vorschläge um den Nutzen zu erhöhen

| **9a** | Könnte man diese Zusammenfassung besser für Sie gestalten?  Wenn es Ihre Aufgabe wäre Änderungen vorzunehmen, was würden Sie ändern?  (Inhalt, Sprache und Terminologie, Darstellung und Formatierung)?   - **Frage noch einmal gezielt nach, falls der/die TeilnehmerIn nur bestimmte Aspekte (z.B. nur Inhalt) kommentiert hat und auf andere gar nicht eingegangen ist.** |
| --- | --- |
|  | |
| **9b** | Hat Ihnen etwas an dieser Zusammenfassung besonders gut gefallen? |
|  | |
| **9c** | Hat Ihnen etwas an dieser Zusammenfassung gar nicht gefallen? |
|  | |

Zugänglichkeit

| **10** | Wo würden Sie erwarten, eine solche Zusammenfassung zu finden? (oder wenn Sie gehört hätten, dass es solche Zusammenfassungen gibt, wie würden Sie bei der Suche nach ihnen vorgehen?)  Haben Sie Vorschläge, wie man sie dem relevanten Publikum leichter zugänglich machen kann? |
| --- | --- |
|  | |

**Sage: Das waren alle Fragen die ich zu der Zusammenfassung habe. Bevor wir zum Schluss kommen habe ich noch ein paar kurze Fragen an Sie.**

# Weiterführende Fragen

Den Test verbessern

| **11** | Haben Sie irgendwelche Vorschläge wie wir diesen User-Test verbessern könnten? Zum Beispiel im Hinblick auf die Information oder Anleitungen, die Sie erhalten haben, etc.? |
| --- | --- |
|  | |

Wortwahl

| **12** | Wir möchten die Zusammenfassung so verständlich wie möglich gestalten, oft ist es jedoch nicht leicht bei technischen Begriffen die richtige Wortwahl zu treffen. Wir haben in der Ihnen vorgelegten Zusammenfassung den Begriff „durchschnittlich“ verwendet. Fanden Sie das verständlich? Würden Sie „mittlere“ bevorzugen? |
| --- | --- |
|  | |

Public Health-relevante Aspekte

| **13** | Sind Angaben zur Finanzierung der Primärstudien und daraus eventuell entstandene Interessenskonflikte ein Punkt, zu dem Sie gerne Informationen in der Zusammenfassung hätten? |
| --- | --- |
|  | |

Zeitaufwand

| **14a** | Was glauben Sie wie viel Zeit Sie bräuchten um diese Zusammenfassung vollständig zu lesen? |
| --- | --- |
|  | |
| **14b** | Könnten Sie sich so viel Zeit in Ihrem Berufsalltag nehmen? |
|  | |

Hemmnisse und Barrieren für die nutzung systematischer reviews

| **15** | Welche Faktoren hindern Sie an der Nutzung systematischer Reviews in Ihrem Berufsalltag? |
| --- | --- |
|  | |

Themen für systematischen Review im follow-up meeting

| **16** | Zu welchen Themen würden Sie gerne die Zusammenfassung eines Reviews lesen? |
| --- | --- |
|  | |

**Sage: Wir sind nun fertig. Vielen Dank für Ihre Teilnahme!**
