# Supplementary material for: Facilitating evidence uptake: development and user testing of a systematic review summary format to inform public health decision-making in German-speaking countries
Source: Health Res Policy Syst. 2018 Jul 9;16:59. doi: 10.1186/s12961-018-0307-z (PMC6038322; doi:10.1186/s12961-018-0307-z)
Supplement: Supplementary file 4 — The revised summary format applied to the Cochrane review on ‘Portion, package or tableware size for changing selection and consumption of food, alcohol and tobacco’ (in English). (DOCX 122 kb) [file 12961_2018_307_MOESM4_ESM.docx]

**Systematic review:
Does the size of portions or packages have an effect on selection or consumption of food, alcohol and tobacco? (2015)**

| **Key messages** | | |
| --- | --- | --- |
| **Background:** | | **Malnutrition and excessive consumption of food, alcohol and tobacco** have been linked to the development of a range of **chronic diseases**.  Food, tobacco and alcohol are offered in different shapes and sizes in shops, vending machines, restaurants and/or bars. It is thus relevant to know whether or not **product size and shape** could be used as potential targets for interventions through public health policy. |
| **Results:** | | This systematic review shows that the **size of portions and packages** as well as the **shape of tableware** affect the **selection and consumption** of food, non-alcoholic beverages and cigarettes.  Effects on selection/consumption of food and non-alcoholic beverages:  Participants **consumed more food and non-alcoholic beverages**, if they were offered **larger portion sizes and packages or specifically shaped tableware** (shorter, wider glasses). |
|  | |  |
|  |  | Effects on selection/consumption of tobacco and alcoholic beverages:  Due to **insufficient evidence**, it is not possible to draw clear conclusions for policy and practice. |
| **Scientific Background:** | | This systematic review includes 72 randomised controlled trials (RCTs), of which 69 focus on food and non-alcoholic beverages and three focus on tobacco. 58 studies were conducted in the USA, while no study was conducted in either Germany, Austria or Switzerland. |

| **Relevance for Public Health Practice** |
| --- |
| Regulatory and legislative frameworks or voluntary agreements with the food industry or shops could influence the availability of smaller portion or package sizes and therefore lead to a health-relevant decrease of consumption of unhealthy food and non-alcoholic beverages. Examples of potential options are:   - Educating people about the effects of portion sizes via food labelling; - Changing the food industry’s pricing practices such that larger portion and package sizes are not cheaper in absolute or relative terms than smaller portions or package sizes; and - Placing of smaller portion sizes in shops closer to consumers or offering smaller portion sizes in restaurants. |

| **Inclusion criteria** | **Characteristics of included studies** | | |
| --- | --- | --- | --- |
| **Participants** | | | |
| Children and adults | 6603 participants overall | - 5182 adults in 55 studies - 1421 children in 17 studies | |
| **Study design** | | | |
| Only randomised controlled trials (RCTs) | 72 RCTs | | |
| **Types of intervention** | | | |
| Studies investigating the influence of alterations in size and/or presentation of food, alcoholic or non-alcoholic beverages and tobacco | Overall: 72 studies  Topics:  69 studies on food  3 studies on cigarettes  0 studies on alcoholic beverages | - Sub-categories:  38 studies on portion size - 12 studies on package size - 6 studies on unit portion size - 16 studies on tableware‘s size or shape | |
| **Outcomes** | | | |
| **Selection** of food, alcoholic or non-alcoholic beverages and tobacco  **Consumption** of food, alcoholic or non-alcoholic beverages and tobacco | Definition of selection: participants could pick from offered products.  Definition of consumption: the actual consumed amount of products. | | 13 studies  65 studies |
| **Setting/Context** | | | |
| No restrictions | Studies were only conducted in high-income countries, predominantly in the USA (n=58). No studies were conducted in Germany, Austria or Switzerland.  50 laboratory studies (e.g. artificial decision situation)  22 real-life condition studies (e.g. restaurants, schools) | | |
| **Time frame** | | | |
| No restrictions | Publication years of studies: 1978-2013. | | |

| **Quality of Evidence Grades (GRADE)** | |
| --- | --- |
| **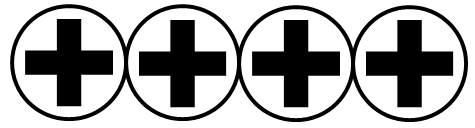** | We are very confident that the true effect lies close to that of the estimate of the effect. |
| **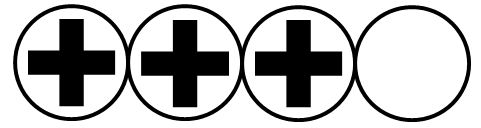** | We are moderately confident in the effect estimate: The true effect is likely to be close to the estimate of the effect, but there is a possibility that it is substantially different. |
| **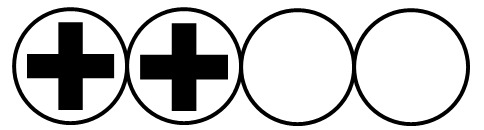** | Our confidence in the effect estimate is limited: The true effect may be substantially different from the estimate of the effect. |
| **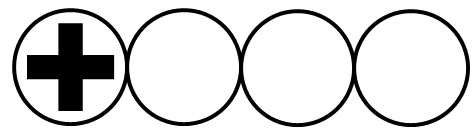** | We have very little confidence in the effect estimate: The true effect is likely to be substantially different from the estimate of effect. |

| **Food:**  **Larger versus smaller-sized portions, packages or tableware** | | | | | |
| --- | --- | --- | --- | --- | --- |
| Population: children and adults  Intervention: larger-sized portion, package, individual unit or item of tableware  Comparison: smaller-sized portion, package, individual unit or item of tableware  Settings: high-income countries, laboratory and real-life settings | | | | | |
| **Results of the study** | | | |  | |
| **Outcomes** | | **Number of participants (independent comparisons)** | **Quality of the evidence**  (GRADE, box) | | **Relative effect**  (95% confidence interval) |
| **Selection** without purchase | | 1164  (13) | **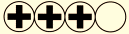** | | **0.42 (0.24 to 0.59**)  standard deviations higher |
| **Interpretation aid**: 13 independent comparisons including 1164 participants showed that participants being offered food in larger-sized portions, packages or tableware (intervention group) selected more food (0.42 standard deviations) than participants being offered smaller-sized portion, package, individual unit or item of tableware.  **Example**: Transferred to the population in the United Kingdom (UK), the mean daily energy intake from food would be 12.4 % or 209 kcal (uncertainty: 119 to 293 kcal) higher. In the subgroup of adults this would relate to an increased mean daily energy intake from food of 10.9 % or 188 kcal (uncertainty: 188 to 403 kcal). In the subgroup of children this would relate to an increased mean daily energy intake from food of 3.8 % or 63 kcal (uncertainty: 27 to 153 kcal). | | | | | |
|  | **Selection** without purchase among **adults** | 782  (9) | **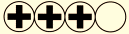** | | **0.55 (0.35 to 0.75)**  standard deviations higher |
|  | **Selection** without purchase among **children** | 382  (4) | **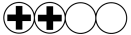** | | **0.14 (0.06 to 0.34)**  standard deviations higher |
| **Consumption** | | 6603  (86) | **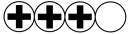** | | **0.38 (0.29 to 0.46)**  standard deviations higher |
| **Interpretation aid**: 86 independent comparisons including 6603 participants showed that participants being offered food in larger-sized portions, packages or tableware (intervention group) consumed more food (0.38 standard deviations) than participants being offered smaller-sized portion, package, individual unit or item of tableware.  **Example**: Transferred to the population in the United Kingdom (UK), the mean daily energy intake from food would be 11.2 % or 189 kcal (uncertainty: 144 to 228 kcal) higher. In the subgroup of adults this would relate to an increased mean daily energy intake from food of 14.3 % or 247 kcal (uncertainty: 215 to 279 kcal). In the subgroup of children this would relate to an increased mean daily energy intake from food of 5.7 % or 95 kcal (uncertainty: 45 to 140 kcal). | | | | | |
|  | **Consumption** among **adults** | 5182  (64) | **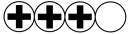** | | **0.46 (0.40 to 0.52)**  standard deviations higher |
|  | **Consumption** among **children** | 1421  (22) | **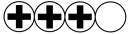** | | **0.21 (0.10 to 0.31)**  standard deviations higher |

| **Non-alcoholic beverages (water and fruit juice):  Shorter, wider versus taller, narrower glasses or plastic bottles (shape)** | | | | | |
| --- | --- | --- | --- | --- | --- |
| Population: children and adults  Intervention: shorter, wider glasses or plastic bottles  Comparison: taller, narrower glasses or plastic bottles  Settings: high-income countries, real-life settings | | | | | |
| **Results of the study** | | | |  | |
| **Outcomes** | | **Number of participants (independent comparisons)** | **Quality of the evidence**  (GRADE, box) | | **Relative effect**  (95% confidence interval) |
| **Selection** without purchase | | 232  (3) | **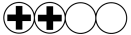** | | **1.47 (0.52 to 2.43)**  standard deviations higher |
| **Interpretation aid**: Three independent comparisons including 232 participants showed that participants being offered beverages in shorter, wider glasses or bottles (intervention group), selected a higher quantity (1.47 standard deviations) than participants being offered beverages in taller, narrower glasses or bottles (comparison group). The volume of glasses and bottles was identical. | | | | | |
|  | **Selection** without purchase among **adults** | 136  (2) | **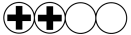** | | **1.03 (0.41 to 1.65)**  standard deviations higher |
|  | **Selection** without purchase among **children** | 96  (1) | **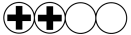** | | **2.31 (1.79 to 2.83)**  standard deviations higher |
| **Consumption** | | 50  (1) | **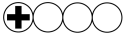** | | **1.17 (0.57 to 1.78)** standard deviations higher |
| **Interpretation aid**: One independent comparison including 50 participants showed that participants being offered beverages in shorter, wider glasses or bottles (intervention group), consumed a higher quantity (1.17 standard deviations) than participants being offered beverages in taller, narrower glasses or bottles (comparison group). | | | | | |

| **Tobacco:**  **Longer versus shorter cigarettes** | | | | |
| --- | --- | --- | --- | --- |
| Population: adults^1^  Intervention: longer cigarettes (with similar amount of tobacco)  Comparison: shorter cigarettes  Settings: high-income countries, laboratory settings | | | | |
| **Results of the study** | | |  | |
| **Outcomes** | **Number of participants (independent comparisons)** | **Outcomes** | | **Number of participants (independent comparisons)** |
| **Consumption** | 108  (6) | **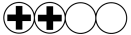** | | **0.25 (0.14 to 0.65)**  standard deviations higher |
| **Interpretation aid:** Six independent comparisons including 108 participants showed that participants being offered longer cigarettes (intervention group), consumed a higher quantity (0.25 standard deviations) than participants being offered shorter cigarettes (comparison group). The amount of tobacco was similar in both kinds of cigarettes.  **Example:** Transferred to the population in the United Kingdom (UK), the mean number of cigarettes smoked per day would be 15.4 % or 2 cigarettes higher with the intervention (uncertainty: 1 to 5 cigarettes)^2^. | | | | |
| ^1^ No eligible studies could be found for children/adolescents.  ^2^ The estimates of the comparison group are based on data from a representative survey in the UK (means and standard deviations of the daily cigarettes smoked). | | | | |

Additional information on the systematic review

| **Differential effects in subgroups and special characteristics** | |
| --- | --- |
| Sex | Meta-analyses didn’t show any differences. |
| Age | Selection and consumption of food: The effect of portion and package size was larger in adults compared to children.  Selection and consumption of non-alcoholic beverages: Insufficient data. |
| Type of food and beverages | Effect sizes were larger in studies of less healthy food and more energy-dense food products. |
| Body-Mass-Index | Insufficient data |

| **Study funding and conflict of interest** |
| --- |
| - Funding source was revealed for the majority of included studies. - There was no evidence of funding by agencies with directly conflicting interests in the results. - There are no conflicts of interest concerning the systematic review authors. |

| **Further information** |
| --- |
| **Systematic review related to the summary**  Hollands GJ, Shemilt I, Marteau TM, Jebb SA, Lewis HB, Wei Y, Higgins JPT, Ogilvie D. Portion, package or tableware size for changing selection and consumption of food, alcohol and tobacco. Cochrane Database of Systematic Reviews 2015, Issue 9. Art. No.: CD011045. DOI: 10.1002/14651858.CD011045.pub2 (http://bit.ly/1Rw43eJ) |
| **Cochrane Glossary (German)**: <http://www.cochrane.de/de/cochrane-glossar>  **Cochrane Public Health**: http://ph.cochrane.org/  **Cochrane Public Health Europe**: <http://ph.cochrane.org/cochrane-public-health-europe> |
| **Contact**  Cochrane Public Health Europe: [cochranepublichealth@ibe.med.uni-muenchen.de](mailto:cochranepublichealth@ibe.med.uni-muenchen.de)  Authors of the summary: Laura K Busert; Margot Mutsch; Christina Kien; Jan M Stratil ; Eva A Rehfuess. |

General information

| **Glossary** | |
| --- | --- |
| **Confidence interval** | Put simply, the confidence interval provides a measure of uncertainty due to the play of chance. More precisely, a confidence interval reflects the extent to which the play of chance may be responsible for a result from a study, such as an effect estimate (or the accuracy of a diagnostic test or the opinions of a population). A 95% confidence interval means that we can be 95% confident that the actual size of the effect is between the lower and upper number given (confidence limit). This means there is a 5% chance that the actual effect is outside of this range. Wider confidence intervals indicate lower precision of the effect estimate; narrower intervals, greater precision. |
| **Effect estimate** | An effect estimate is a statistical measure indicating the most likely size of an intervention effect. |
| **GRADE** (=Grading of Recommendations, Assessment, Development and Evaluation) | GRADE offers a systematic approach for assessing and communicating the certainty of evidence and going from evidence to recommendations or decisions. After summarising and assessing the best available evidence, GRADE offers distinct criteria to evaluate the quality of the evidence (confidence in the effect estimate) such as: study design, risk of bias, imprecision, inconsistence, indirectness and strength of the effect. |
| **Independent comparison** | An independent comparison considers differences in the occurrence of an outcome in the intervention and control group. Independent refers to the fact that within a study, different comparisons can be conducted that may or may not be independent from each other. |
| **Outcome** | In studies investigating the effects of interventions, outcomes are measures of health or disease (e.g. obesity, quality of life), behaviours (e.g. smoking, drinking), or other potential benefits or harms of the interventions. |
| **p-value** | The p value indicates the probability (ranging from 0 to 1) that the results observed in a study could have occurred by the play of chance. More precisely, for intervention effect estimates, the p-value is the probability that the results observed in a study could have occurred by the play of chance, if the intervention actually had no impact on the outcome. |
| **Randomised controlled trial (RCT)**  **Synonyms:**  **randomised study, randomised trial** | Put simply, randomised controlled trials are a category of [studies](http://getitglossary.org/term/study) comparing two or more interventions in which [random allocation](http://getitglossary.org/term/allocation,%20random) is used to assign [participants](http://getitglossary.org/term/participants) to [intervention comparison groups](http://getitglossary.org/term/treatment%20comparison%20group). More precisely, randomised studies, commonly called randomised trials, are an intervention [comparison](http://getitglossary.org/term/treatment%20comparison) in which two or more interventions, possibly including a [placebo](http://getitglossary.org/term/placebo) or withholding an intervention, are compared after random allocation of participants to intervention comparison groups. Random allocation ensures that each participant has a known (usually an equal) chance of being assigned to any given group. This results in intervention comparison groups that are similar in terms of [prognostic variables](http://getitglossary.org/term/prognostic%20variable), whether or not these have been recognised. Thus, there is generally a lower risk of allocation bias in randomised studies than there is in non-randomised studies. |
| **Relative effect** | Relative effects are ratios of outcome measures between intervention and comparison groups in a study. Put differently, measures of relative effect express the outcome in one group relative to that in the other. For example, if death is the outcome and 6% (6 per 100) in the intervention group die and 10% (10 per 100) in the comparison group die, the risk ratio (a measure of the relative effect) is 6/10 = 0.60. |
| **Standard deviation** | The standard deviation is a statistical measure which is used to quantify the amount of variation of a set of data values. In this summary format, a low standard deviation indicates that the data points tend to be close to the effect estimate, while a high standard deviation indicates that the data points are spread out over a wider range of values. |
| **Systematic review** | Put simply, a systematic review is a summary of [studies](http://getitglossary.org/term/study) addressing a clear question, using systematic and explicit methods to identify, select, and critically appraise relevant studies, and to collect and analyse [data](http://getitglossary.org/term/data) from them. More precisely, systematic reviews of [research](http://getitglossary.org/term/research) [evidence](http://getitglossary.org/term/evidence) use scientifically defensible, explicit methods to reduce [bias](http://getitglossary.org/term/bias) (systematic error) and, if appropriate and possible, [meta-analysis](http://getitglossary.org/term/meta-analysis) to reduce the [play of chance](http://getitglossary.org/term/chance,%20play%20of). |
| **Uncertainty** | In the context of this summary review format, the term uncertainty refers to the confidence limit. A 95% confidence interval means that we can be 95% confident, that the actual effect size is between the lower and upper number given. |

Most glossary terms were adopted/adapted from [www.getitglossary.org](http://www.getitglossary.org).
